# Unexamined Endocrine Disruption? Pesticides Inhibit Prostaglandin Activity

**DOI:** 10.1289/ehp.124-A76

**Published:** 2016-04-01

**Authors:** Lindsey Konkel

**Affiliations:** Lindsey Konkel is a New Jersey–based journalist who reports on science, health, and the environment.

Androgens are widely recognized as important drivers of male sexual development in the fetus.^1^ Some endocrine-disrupting chemicals have been shown to impact male sexual differentiation by affecting androgen production or blocking androgen receptors in target tissues.^2^ Comparatively little attention has been paid to the role that ubiquitous biologically active lipids called prostaglandins play in these developmental processes, although studies have found that prostaglandins help initiate male genital development.^3^^,^^4^^,^^5^ In this issue of *EHP*, researchers show that several structurally different endocrine-active pesticides suppress prostaglandin synthesis and signaling in mouse cells.^6^

“The research provides support for a new modality of endocrine disruption via prostaglandin inhibition,” says senior study author Andreas Kortenkamp, a toxicologist at Brunel University in London. This study is thought to be the first to test pesticides for these effects.

**Figure:**
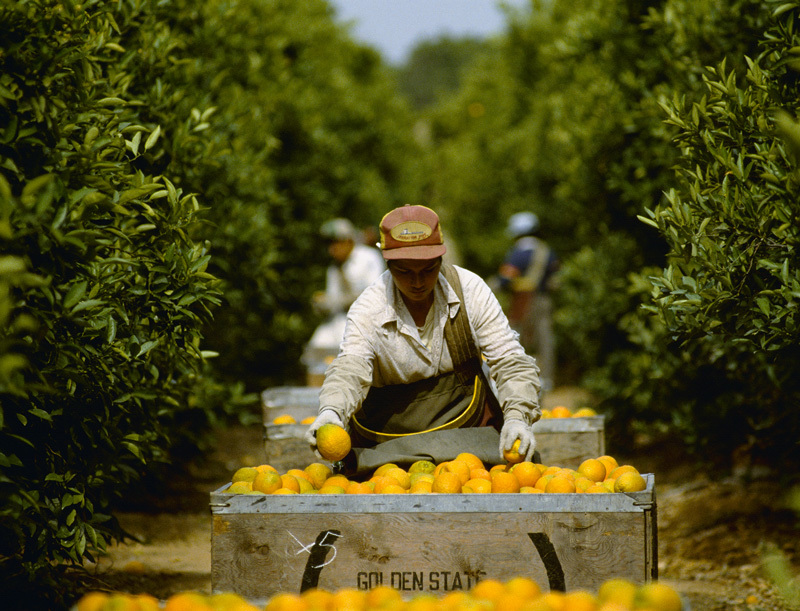
OPP is applied to citrus fruits and other crops after harvest to prevent spoilage. The general population is thought to consume levels of OPP well below recommended limits, although workers likely receive greater exposures.^12^ It is unknown whether OPP causes human health effects. © David Thurber/Corbis

Prostaglandins serve many purposes—from the promotion and resolution of the immune system’s inflammatory response^7^ to male sexual development *in utero*.^5^ Previous studies have suggested that synthesis of prostaglandin-D2 (PGD2) may serve as a back-up mechanism for androgen-driven differentiation of Sertoli cells^8^—testicular cells that play an important role in the development and production of sperm.

A variety of mild analgesics (including acetaminophen, aspirin, and ibuprofen) have been shown to suppress PGD2 synthesis in human and rodent cell lines.^9^ In one study, self-reported use of aspirin, acetaminophen, or ibuprofen during pregnancy was associated with cryptorchidism in some boys but not others, and experimental findings for rats indicated shorter anogenital distance with exposure to acetaminophen or aspirin.^10^ According to the American Academy of Family Physicians, acetaminophen is generally considered safe for use during pregnancy, but this assessment is based on limited evidence.^11^ Pregnant women are advised to use aspirin and ibuprofen only under a doctor’s supervision.^11^

In the current study, Kortenkamp and colleagues tested the ability of 24 pesticides commonly used in the European Union to suppress PGD2 synthesis in juvenile mouse Sertoli cells. Fifteen of the pesticides, including imidacloprid, cypermethrin, and *ortho*-phenylphenol (OPP), inhibited PGD2 synthesis in a concentration-dependent manner.^6^

OPP, an antimicrobial agent used as a fungicide and sanitizer, was the most potent pesticide tested, suppressing PGD2 synthesis at a concentration of 175 nM. This made it nearly as potent as ibuprofen (used as a positive control), which suppressed PGD2 synthesis at a concentration of 128 nM.^6^ “We were really surprised by the potency of some of these pesticides, namely OPP,” Kortenkamp says.

Molecular modeling suggested that OPP and other pesticides may interfere with prostaglandins by inhibiting cyclooxygenase-1 and cyclooxygenase-2—the enzymes responsible for prostaglandin synthesis—in a manner similar to ibuprofen. Eleven of the 15 pesticides shown to suppress prostaglandin synthesis also acted as antiandrogens, chemicals that interfere with androgen production by blocking the androgen receptor.^6^

The new paper is important, because it shows that several of the investigated pesticides may obstruct both the androgen and prostaglandin signaling pathways, says David Kristensen, a toxicologist at the University of Copenhagen in Denmark. “The implications of this collective dual-hit scenario are not yet clear,” says Kristensen, who was not involved in the current study.

It’s difficult to predict likely effects of these pesticides on humans from cell culture studies alone, Kortenkamp says. The next step for his team is to identify priority chemicals for testing in animal models. Factors such as the rate at which a chemical enters the body and how rapidly it is metabolized and excreted can help to predict chemicals that would be expected to show activity in fetal tissue at experimental doses.
